# Low Rate of Between-Population Seed Dispersal Restricts Genetic Connectivity and Metapopulation Dynamics in a Clonal Shrub

**DOI:** 10.1371/journal.pone.0050974

**Published:** 2012-11-29

**Authors:** Laura Merwin, Tianhua He, Byron B. Lamont, Neal J. Enright, Siegfried L. Krauss

**Affiliations:** 1 Department of Environment and Agriculture, Curtin University, Perth, WA, Australia; 2 Kings Park and Botanic Garden, Botanic Gardens and Parks Authority, Perth, WA, Australia; 3 School of Environmental Science, Murdoch University, Perth, WA, Australia; 4 School of Plant Biology, University of Western Australia, Perth, WA, Australia; 5 Department of Ecology and Evolution, University of Chicago, Chicago, Illinois, United States of America; CNR, Italy

## Abstract

Clonal species normally have low seed production, low recruitment rates and long lifespans, and it is expected that the rates of long-distance dispersal (LDD) of seeds will be low as well. *Banksia candolleana* is a clonal shrub in Mediterranean-type, fire-prone sclerophyll shrublands of southwestern Australia, whose reproductive biology and population dynamics contrast with those of co-occurring nonclonal congeneric species, all of which are restricted to a mosaic of sand dunes set within a matrix of inhospitable swales. Using microsatellite markers, we genotyped 499 plants in all 15 populations of *B. candolleana* within a 12-km^2^ area, assessed population genetic differentiation, and quantified the effective rate of interpopulation seed dispersal through genetic assignment of individuals to populations. We measured life history, reproductive and demographic attributes, and compared these with two co-occurring *Banksia* species, a non-clonal resprouter and a nonsprouter. *B. candolleana* has much higher levels of population genetic differentiation, and one-third the rate of interpopulation seed migration, as the other two species (2.2% vs 5.5−6.8% of genotyped plants inferred to be immigrants), though distances reached by LDD are comparable (0.3−2.3 km). The low rate of interpopulation dispersal was supported by an analysis of the age structure of three populations that suggests a mean interdune migration rate of <800 m in 200 years, and 60% of suitable dunes remain uninhabited. Thus, *B. candolleana* has poor properties for promoting long-distance dispersal. It is unclear if these are idiosyncratic to this species or whether such properties are to be expected of clonal species in general where LDD is less critical for species survival.

## Introduction

Landscape genetics addresses how landscape elements, environmental factors and species life-histories influence the process and pattern of genetic connectivity and the spatial distribution of genetic variation within and among populations [1], [2], [3]. Genetic connectivity among plant populations is achieved through the movement of pollen and seeds, but especially through the dynamics of seed dispersal [4], [5]. Most seeds are dispersed within the vicinity of the maternal plant, but long-distance dispersal (LDD) vectors may take seeds into new habitats or different populations. Though rare, these events are disproportionally important for species dynamics at the landscape scale [4], [6]. LDD of seeds, defined by Nathan et al. [7] as dispersal to a distance at least 100 times plant height, plays a crucial role in metapopulation dynamics through colonisation of new habitats and/or recolonisation following local extinction [8]. Indeed, dispersal may be considered a bet-hedging strategy, allowing metapopulation persistence when any single habitat patch is only transiently favorable [9].

Understanding the landscape, environmental and life-history constraints affecting the ability of plants to migrate is increasingly important as species respond to global environmental change [3]. As population fragmentation, environmental heterogeneity, and impacts on LDD vectors increase in many areas, understanding the dispersal dynamics of plants is vital to shaping effective conservation strategies and predicting the magnitude of landscape change for affected populations [3], [10]. LDD modeling is severely constrained by current data limitations [6]. Efforts to quantify LDD events are difficult due to the rare nature of these events and their dependence on non-standard vectors and/or chance occurrences [4], [11]. However, given an appropriate set of conditions [1], genetic assignment tests using molecular markers have had much success in identifying immigrants and their source, quantifying LDD rates, and generating significant detail about the tail of the seed dispersal curve [12], [13], [14], [15]. When combined with demographic data, these genetic approaches provide powerful insights into population connectivity [16].

The South West Australian Floristic Region (SWAFR) comprises 300 000 km^2^ of sclerophyll forests, woodlands, and shrublands and is internationally recognized as a biodiversity hotspot [17]. Characterized by a Mediterranean-type climate with hot, dry summers and cool, wet winters, the region is periodically subject to wildfire and severe drought. Plant species in the SWAFR are classified by life-history categories that are defined by fire response. Nonsprouters (NS) are killed by fire and produce a new generation from seeds after each fire event. Resprouters (RS) undergo vegetative regrowth after fire from structures insulated from fire heat, as well as producing seedlings immediately post-fire. Because fire response influences many critical plant characteristics, the NS vs. RS dichotomy is often considered a key determinant of Mediterranean plant ecology [18], [19]. The NS vs. RS dichotomy extends to life-history traits including seed set and viability, seedbank size and seedling survival, all of which are lower in resprouting species [19], [20], [21]. These differences are often viewed as the result of a resource mediated tradeoff between resprouting (growth of vegetative structures) and seed production [22], [23], although there is little empirical support [24].

Both RS and NS shrubs and trees in the genus *Banksia* are dominant plant community members in much of the SWAFR. Three previous studies measured long-distance dispersal of seed in *B. hookeriana* (NS) [13], [15] and *B. attenuata* (RS) [14]. In accordance with the general NS/RS dichotomy, *B. attenuata* produces and stores far fewer viable seeds (10%) than *B. hookeriana* [20], [22]. In addition, *B. attenuata* produces seeds twice the weight of *B. hookeriana* (101 vs 45 mg, [25]) that have a higher terminal velocity (3.49 vs 2.83 ms^−1^, [25]). *B attenuata* plants are also shorter (97 vs 147 cm at 12 y after fire, [T. He, unpublished data]). Based on these trait values, it was expected that LDD would be much lower for *B. attenuata* than for *B. hookeriana* [9], [14]. However, rates and distances LDD were similar between the two species. For co-occurring metapopulations, 5.5% of *B. attenuata* individuals genotyped were inferred as immigrants (microsatellite markers) compared with 6.8% (AFLP markers) and 5.5% (microsatellite markers) of *B. hookeriana*, with a mean/maximum detected dispersal distance of 1.4/2.6 km and 2.0/2.5 km respectively [13], [14], [15]. Thus, seeds of this NS/RS pair appear to be equally mobile and capable of long-distance dispersal, despite the differences in seed production and size.

In order to further assess the impact of life-history traits on LDD and genetic connectivity, we chose a congeneric species with traits representing the extreme of the resprouting class. Here, we examine *Banksia candolleana*, a (typical) clonal resprouter with seedling recruitment rates and longevity differing markedly from *B. attenuata. B. candolleana* is an extremely long-lived (up to an estimated 1200 years, see Methods), outcrossing, creeping shrub that co-occurs with *B. attenuata* and *B. hookeriana* in the SWAFR. Although its seed bank is comparable with *B. attenuata* (due to its larger crown), initial postfire seedling establishment is only 5−25% of *B. attenuata* and seedling survival over the first summer-autumn postfire is negligible [20]. *B. candolleana* also produces much larger seeds than *B. attenuata* (213 mg vs 101 mg) that are held close to the ground and are hidden within the interior of the ground-hugging crown. Although winged like those of other banksias, *B. candolleana* seeds are primarily gravity dispersed. Based on these characteristics, we hypothesized that *B. candolleana* would experience much lower levels of LDD than the co-occurring *B. attenuata* and *B. hookeriana*.

## Materials and Methods

### Study Area and Sampling

**Figure 1:**
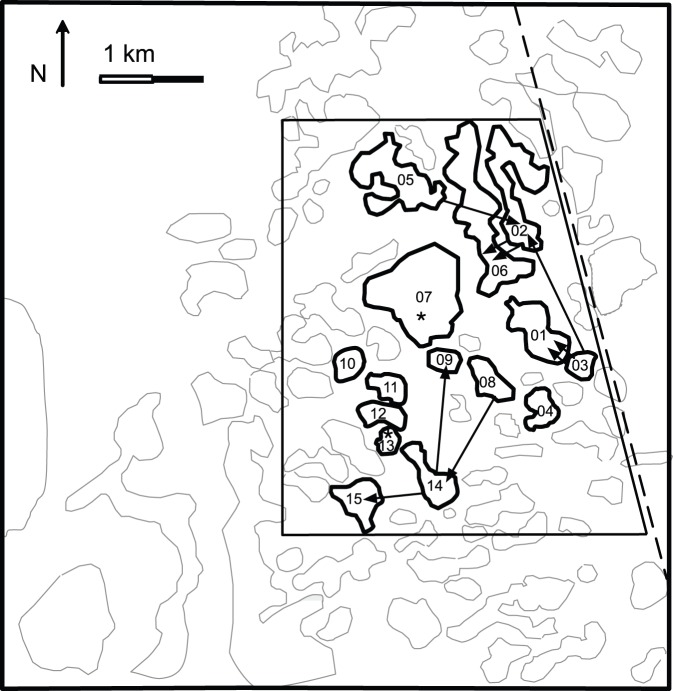
Location of *B. candolleana* populations (numbered), showing that populations are geographically restricted to dune crests and separated by intervening dune swales. Arrows indicate seed dispersal events. Asterisks in population BC07 and BC13 indicate immigrant for which the most likely source population was not determined.

The study site was located on the Eneabba Plain in the SWAFR, in a 3×4 km area centered at E115°13′54″, S29°44′29″. This region has a dry Mediterranean-type climate and a mean fire interval of 17–28 years since 1970 [26]. The sandplain is characterized by a dune and swale system, with plant communities differing between dune crests and swales (as a result of shallower soils and consequent lower water availability in the swales). *Banksia attenuata*, *B. candolleana* and *B. hookeriana* are dominant species of the dune crest shrub/tree communities and are not present in the intervening swales (separating dunes by 65−850 m, Fig. 1). As for *B. hookeriana* and *B. attenuata*, we considered *B. candolleana* to exhibit a metapopulation structure in the area, viewed as an assemblage of local populations that are discrete geographic entities with some interaction via gene flow [27]. We visited all 40 dunes in the study area, identified 15 populations of *B. candolleana*, and sampled 40 individuals from each population, with the exception of dune BC05, where population size limited sampling to 25 individuals. Sampled individuals were at least 20 m apart to ensure the sampling of unique genets rather than ramets of the same clone. We harvested healthy leaves and stored them in zip-lock bags on ice until they could be frozen at −80°C.

### Genotyping and Genetic Structure Analysis

Total genomic DNA was extracted following Doyle [28] from 0.5–0.8 g of leaf material with the addition of 5 M potassium acetate and sodium chloride as purification steps. Quantity and quality of extracted DNA were assessed by visualization on 2% agarose gel and Nanodrop (Thermo Fisher, Wilmington, DE). Eleven microsatellite primer pairs were used to genotype 592 samples as described in Merwin et al. [29]. We eliminated 93 samples for which genotyping failed at more than one locus, and continued analysis with the remaining 499 individuals. This reduced sample size per population to 33−39 individuals per population, with one at 24 (Table 1).

**Table 1:** Sampled *Banksia candolleana* population characteristics.

Population	#Samples	Est PopSize	Density(ha^−1^)	N_a_	H_e_	Immigrants
BC01	37	400	19	8.1	0.73	0
BC02	36	120	9	9.0	0.77	2
BC03	37	120	17	8.1	0.75	1
BC04	24	30	4	6.7	0.68	0
BC05	36	500	10	8.2	0.73	1
BC06	37	150	43	7.9	0.76	2
BC07	36	4000	74	8.5	0.73	1
BC08	36	210	20	7.0	0.71	0
BC09	33	200	20	8.0	0.76	1
BC10	38	50	8	6.4	0.64	0
BC11	34	40	8	7.9	0.75	0
BC12	35	100	34	8.0	0.71	0
BC13	39	100	53	8.0	0.70	1
BC14	33	50	6	6.5	0.69	1
BC15	38	100	6	7.8	0.72	1
Average	32	410	22	7.7	0.72	0.7

.# = number; Est Pop = Estimated population; N_a_ = mean number of alleles per locus; H_e_ = mean expected heterozygosity; Immigrants = number of samples assigned to a population other than that from which it was sampled. All but 2 of these immigrants were confidently assigned to a single source population.

The data were initially analyzed for population genetic parameters of number of alleles (N_a_) and expected heterozygosity (H_e_), and population differentiation was estimated using both F_ST_ and Analysis of Molecular Variance (AMOVA) using GenAlex 6.0 [30]. Deviations from Hardy-Weinberg Equilibrium (HWE) and linkage equilibrium (LE) were tested using GenePop [31], and where consistent deficits of heterozygotes were detected, the possible presence of null alleles was tested using Microchecker [32]. Since *B. candolleana* is clonal, an analysis of clonality was conducted in GenClone2 [33] so that possible ramets from the same clone could be excluded from further population assignment analysis.

### Population Assignments

We conducted population likelihood assignment tests to infer the seed source population for each sampled individual, following procedures applied previously for *B. attenuata* and *B. hookeriana* (14–15). Microsatellite loci that deviated from HWE and LE were excluded from assignment analysis. We assumed that all alleles were shared across candidate source populations. GeneClass2 [34] was first used to calculate the probability that an individual is a resident (i.e. not a first generation migrant) in the population from which it was sampled. The Paetkau *et al.* [35] Monte Carlo resampling method with 10,000 resampled individuals was used, and an individual with *P*<0.001 was excluded from classification as a resident. Following Rannala and Mountain’s likelihood Bayesian-based method [36], GeneClass2 [34] was used to estimate allelic frequencies for each population, and the likelihood of originating from each source population was calculated for each individual, resulting in a score (percentage) being given for each population as the source of each sample.

An assignment was accepted as unambiguous when the difference (δ) between the largest and the second largest log-likelihood was above a predetermined threshold stringency level. Adopting too low a level of stringency (i.e. δ near 0) increases the risk of falsely assigning individuals as immigrants due to, for example, inter-population pollen flow [37]. Too high a level of stringency (e.g. δ = 3, i.e 10^3^ times more likely to originate from the population with highest log-likelihood than the second highest log-likelihood) limits the assignment efficiency in terms of the fraction of individuals that can be assigned to any population [38]. In addition, the likelihoods of a particular individual being a resident or an immigrant are not equal, as LDDs are much rarer events than local dispersal events [6], as demonstrated by the low values (5.5% and 6.8%) of immigrants detected for two sympatric congeners [13], [14], [15]. Therefore, we applied a stringency level of δ = 1.0 to accept an individual as an immigrant, i.e. a multilocus genotype had to be at least 10 times more likely to be assigned to a source population other than the (home) population from which it was sampled [38]. This criterion excluded inter-population pollen dispersal events and early crosses between residents and non-residents, as the resulting genotypes would be intermediate between the two parental genotypes and not be confidently assigned to a single population [13].

Our approach provides a stringent and conservative method for identifying seed immigrants but does risk underestimating migration rate [39]. Our choice of the value (*P*<0.001) at which we exclude an individual as a resident minimizes the probability that local residents are wrongly identified as immigrants, but also potentially underestimates migration rates. As a consequence, we further tested seed migration rate by implementing an assignment-based method that jointly estimates individual population membership and recent migrant proportions to obtain an unbiased migration rate, called BayesAss [40].

### Life-history Traits and Demographic Attributes

Life-history traits and demographic and morphological attributes were collected for the three *Banksia* species. Available information was collated from >30 years of demographic/life-history studies [20], [41], [42], [43], [44] and supplemented with new data as required. These data included fire-caused and inter-fire mortality, canopy seed store (i.e. seed bank of serotinous cones), postfire recruitment rates and recruitment efficiency (number of recruits per plant after fire divided by number of seeds stored per plant at time of fire). Plant height and crown width (in two dimensions) were obtained for 140 mature plants of *B. candolleana* in an area last burned 12 years ago and spanning a number of low sandy dunes to which the banksias are restricted. Plant density was determined as the number of plants per dune divided by dune size estimated from satellite imagery [45]. Seed production (per ha) was calculated as fecundity per plant × population density (ha^−1^). Mature resprouts of *B. candolleana* (and *B. attenuata*) begin producing seeds again 2–3 years after fire and return to pre-fire height within 4–5 years. *B. hookeriana* recruits from seed after fire, takes longer to reach maturity (4–5 years) and continues to increase in height with age. By the time stands are 12 years old they are capable of carrying fire again and all three species have reached a large size and accumulated a serotinous seed bank over 7–10 years.

Lifespan was estimated as maximum possible age of an individual plant. Plants of *B. candolleana* on three large dunes (Dune 05, 07, 15), matched by climate, fire regime and soil-substrate type, were surveyed. The three dunes differ in size but are similar in height (surrogate for soil depth/water availability) and possess the same set of dominant plant species [45]. There was no detectable difference in height of co-occurring *B. hookeriana* and *B. attenuata*, two dominant species on the dunes [20], confirming growing conditions were similar. For *B. candolleana*, below-ground parts of plants were unearthed and the mean length of rhizome produced between fires was measured (7.5±2.5 cm; indicated by horizontal extension of rhizome at the base of vertical branches produced in response to fire). This was divided into the maximum basal radius of clones (n = 140) and multiplied by the estimated mean fire interval (∼15 y, [46]) to give an estimated age for each clone (the maximum width recorded was 1030 cm). A gamma distribution was fitted to the data and extrapolated, giving a maximum estimated longevity of 1200 y (12±4 (SD) m in diameter). Where clumps with otherwise identical leaf and stem morphology were separated by >2 m they were treated as separate plants (genets) but it is possible some were in fact ramets, so that, together with the likelihood of longer fire intervals in the long term as indicated by data for the last 40 y [26], 1200 y must be considered conservative. Data for each of the three populations were put into age (100-cm interval size) classes and used to estimate the time the latest bout of colonisation of the dune occurred (the age of the oldest clone). The right-hand sides of the frequency data were regressed against size classes using negative exponential or logarithmic curves (whichever gave the better fit) in Microsoft Excel version 14.2.3 and the age of the oldest clone was identified by extrapolation to one.

The 3×4 km study area was surveyed for occurrence of the three *Banksia* species. For each species, potential habitat occupancy was calculated as the fraction of dunes with the species present. Fresh seeds were removed from their follicles after heating to open them, 20 seeds of each species with undamaged wings weighed individually, and their terminal velocity determined by noting the time to fall a distance of 5.5 m. Results were corrected for initial free fall after Clements ([47], see equation 4). Ten plants of each of the three co-occurring species at a representative site 16 y since fire were assessed for the height of all cones per plant (mean of 10 plants for *B. candolleana* and *B. attenuata*, and 20 for *B. hookeriana*) were held above the ground (release height for seeds) and their vertical distance to the edge of the crown as an index of obstructions to their potential postfire uplift by wind.

One issue was whether limited pollen flow might have contributed to the higher between-population molecular variance of *B. candolleana* compared with the other two species (see Results). Types of pollinators, levels of pollen production on an inflorescence and plant basis, estimated pollen load per pollinator, and extent of coflowering within plants and populations for the three species, were obtained from the pollinator literature [25], [48], [Enright and Lamont, unpublished], [Lamont, pers. observ]. Since most observations were qualitative (except for *B. hookeriana*), the data were ranked and averaged to give an estimate of relative interpopulation pollen flow for the three species.

## Results

### Genetic Variation and Differentiation

We scored eleven microsatellite primer pairs that amplified 177 alleles (7 to 29 alleles per primer pair, average 16.1 per locus, SD = 7.6) across 499 individuals from 15 populations. The mean number of alleles (N_a_) amplified per locus ranged from 6.4 in population BC05 to 9.0 in population BC02 with an average of 7.7. The expected heterozygosity ranged from 0.638 (BC15) to 0.768 (BC02). A significant and large deficit of observed heterozygotes from HW equilibrium expectations was found for the loci D10 and A6 for all but one population (overall *F*_IS_ = 0.48 and 0.68, respectively). The possibility of null alleles at these two loci was identified by analysis with Microchecker. Evidence for highly significant linkage disequilibrium was also obtained for 10 of 55 pairwise locus comparisons, 4 of which involved the loci D10 or A6. The AMOVA for all eleven loci partitioned 85% of the total observed genetic variation within populations and 15% among them, both of which were significantly >0 (*P*<0.001). Overall F_ST_ = 0.11, and pairwise population F_ST_ ranged from 0.01 to 0.11, with a mean of 0.06. Analysis of clonality confirmed that all sampled individuals were different genets.

### Population Assignments

Population assignment tests were conducted with the 9 loci in HW equilibrium (all but D10 and A6). All 499 samples generated at least one probability of population assignment >0.001 for the sampled populations, suggesting that all individuals originated from one of the sampled populations. Eleven individuals from 9 populations were determined to be immigrants (Table 1), i.e. assigned to a source population other than the one from which they were sampled with a δ>1.0 (Table 2). The result gives an immigration rate for *B. candolleana* in the metapopulation of 2.2%. Among the 11 immigrants, δ between the first and second most likely populations was >1.0 for nine samples, allowing an unambiguous determination of source population. For the remaining two samples, the δ between the first and second most likely populations was <1.0, while the δ between the second most likely and home populations (third most likely) was >1.0. Thus, although these samples were clearly immigrants, they could not be unambiguously assigned to a source population (Table 2). The mean distance travelled by the nine immigrants with an unambiguous source population identified was 1.1 km with a range of 0.3−2.3 km (Fig. 1, Table 3). Seeds dispersed in most directions of the compass, except due south, with no clear bias in other directions. BayesAss provided an unbiased between-population migration rate of 0.022, with a 95% credible range between 0.011 to 0.033.

**Table 2:** Immigrants, and their source populations, inferred by population assignment test.

ID	Sink population	Most likely source population (score)	Second most likely source population(score)	δ^1^	δ^2^
269	BC02	BC05 (99.6%)	BC02 (0.3%)	2.483	–
302	BC02	BC03 (86.6%)	BC07 (6.7%)	1.112	1.128
053	BC03	BC01 (91.5%)	BC03 (8.4%)	1.038	–
333	BC05	BC07 (80.7%)	BC03 (6.9%)	1.065	1.085
361	BC06	BC02 (81.8%)	BC06 (7.6%)	1.032	–
364	BC06	BC02 (98.8%)	BC06 (0.6%)	2.194	–
212	BC09	BC14 (100.0%)	BC13 (0.0%)	7.719	8.119
220	BC14	BC08 (90%)	BC09 (9.0%)	2.202	4.228
476	BC15	BC14 (100.0%)	BC15 (0.0%)	4.859	–
328*	BC07	BC09 (48.9%)	BC03 (33.8%)	0.004	1.264
498*	BC13	BC15 (64.2%)	BC12 (0.3%)	0.279	1.501

δ^1^ is the difference in log likelihood between the most likely source population and the second most likely source population. δ^2^ is the difference in log likelihood between the most likely source population and home population when the home population was not the second most likely source population.

* Two potential source populations (δ values between the most likely and the second most likely are smaller than 1.0, while δ values between the second most likely source population and home population are greater than 1.0).

**Table 3:** *F*_ST_, between population variance (AMOVA) and (effective) inter-population seed dispersal rates and the mean among-dune dispersal distance for *B. candolleana* compared with two other co-occurring banksias.

Species	*F*_ST_	AMOVA (Φ_ST_)	LDD rate (% population)	Mean among-dune dispersal distance ± sd and range (km)	Source
*B. candolleana*	0.11	0.15	2.2	1.1±0.9 (0.3−2.3)	This study
*B. attenuata*	0.02	0.02	5.5	1.4±0.7 (0.2−2.6)	[14]
*B. hookeriana*	0.08	0.08	6.2	1.1±0.7 (0.3−3.3)	[13], [15]

### Life-history Traits and Age of Populations

**Figure 2:**
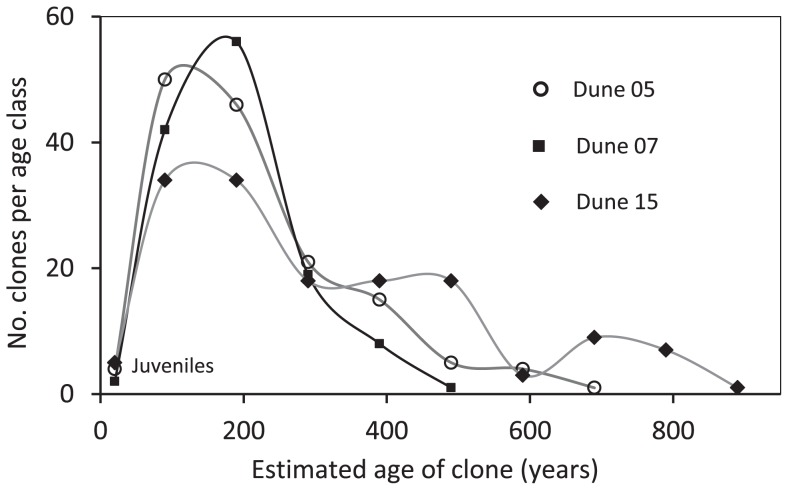
Estimated age distribution of *B. candolleana* clones in three populations (dunes) as located in Fig. 1. Juveniles are <40 y old. A colonisation event is identified as age of the oldest plant (extrapolated by curve fit) in the population.

Of the three species, *B. candolleana* occupied the fewest dunes with the lowest density per dune; it took longest to reach maturity, with negligible post- and interfire mortality and recruitment (Table 4). This species had the smallest seed bank and recruitment efficiency was an order of magnitude less than the other two species. It had the largest seeds but a similar terminal velocity on the faster side of the other species. Plants were the widest and shortest, with cones located closest to the ground but with the greatest distance for released seeds to reach beyond the crown after fire. Most clones (62.4%) were in the 50−250 y age classes, with juveniles (yet to fruit) comprising 2.6%, tapering to one plant estimated at 490 y (negative exponential fit) in population 7 (one sampled plant actually at 485 y), 690 y (negative exponential) in population 5 (one sampled plant at 640 y) and 940 y (negative logarithmic) in population 14 (three sampled plants at 840 y), all with R^2^>85% (Fig. 2). Extrapolation of the entire data set collectively took the oldest clone to 1200 y.

**Table 4:** Life-history traits and demography for *B. candolleana* and two other co-occurring banksias.

Traits	*B. candolleana*	*B. attenuata*	*B. hookeriana*
Fire response	Resprouter −rhizomatous(clonal)	Resprouter −lignotuberous(non-clonal)	Killed(non-clonal)
Habitat occupancy (% of available)	38	100	45
Population density (plants ha^−1^)	48	330	827
Crown width ± se (cm)	208±24	126±18	187±15
Plant height ± se (cm)	76±5	97±6	147±8
Time to 50% with fruits (year)	40	25	5
Lifespan (year)	1200	300	40
Fire-caused mortality (%)	0	3	100
Annual mortality 10−15 y postfire (%)	0.2	1	5
Seed bank (seeds plant^−1^)	32	55	370
Recruits per parent	0.007	0.06	>1.00
Recruitment efficiency (recruits seed^−1^×10^−3^)	0.22	1.09	>2.70
Seed mass ± sd (mg)	213±50	101±12	45±6
Seed terminal velocity ± sd (m s^−1^)	2.30±0.86	3.14±0.91	2.64±0.71
Seed release height ± sd (cm)	22±7	105±9	105±20
Seed position (distance to edge of crown ± sd) (cm)	56±9	15±4	29±11

Data were obtained or recalculated from [20], [41], or authors’ unpublished data, or collected as described in Methods.

## Discussion

Our landscape genetic analysis of the clonal species *Banksia candolleana* involved the same study site, researchers and methodology as for two other *Banksia* species [14], [15]. Therefore, our results are directly comparable in at least a relative sense, independent of the absolute accuracy of the population assignments [49]. Concordance with independent analyses of the data, however, suggests that our estimates of migration rate are accurate. Our results thus enable a comprehensive assessment of the factors influencing dispersal potential and connectivity among populations of the three species. These suggest a need to explain the higher genetic differentiation between populations of *B. candolleana* (F_ST_ = 0.11, Φ_ST_ = 0.15), compared with the other two study species, especially the resprouter, *B. attenuata* (F_ST_ = 0.02, Φ_ST_ = 0.02). Collating data we have on pollinators and relative pollen production and transport (Table 5), interpopulation pollen flow is expected to be only marginally less successful for *B. candolleana* than for *B. attenuata*, with both far less efficient than *B. hookeriana*, a highly successful bird-pollinated species that has strong evidence of interpopulation pollen flow [25], [48]. Therefore, differences in genetic structure can essentially be attributed to an effective LDD rate among seeds in *B. candolleana* that is one-third that of *B. attenuata* (Table 3). However, LDD seed dispersal distances were similar. Restricted interpopulation seed dispersal is likely then to be an important driver of the differences in among population genetic differentiation observed between these species, giving more permanence to any genetic bias among the founders. Weaker landscape genetic structure observed with *B. attenuata* and *B. hookeriana* is a consequence of much higher rates of seed LDD (and possibly pollen flow in the case of *B. hookeriana*, Table 5), and more frequent population turnover rates.

**Table 5:** Pollinators (most important in bold) and relative pollen and co-flowering levels (1 = highest rank) and likelihood of interpopulation pollen flow (Σ/mean for all processes).

Species	Pollinators	Pollen/inflorescence	Pollen/flowering plant	Pollen load/pollinator	Coflowering/plant	Coflowering/population	Interpopulation pollen flow (Σ/6)
*B. candolleana*	Birds, **mammals**, insects (2)	3	2	2	3	3	2.5
*B. attenuata*	**Birds**, **mammals**, insects (2)	2	3	3	2	2	2.3
*B. hookeriana*	**Birds** (1)	1	1	1	1	1	1

See Methods for more details.

To explain the distances reached by *B. candolleana* interpopulation immigrants, the uplift and transport processes proposed for the other two species (wind vortices, cockatoos; [13], [14]) appear to apply to *B. candolleana,* as seed structure is identical and terminal velocities show little difference (Table 4). Postfire damaged follicles of *B. candolleana* [consistent with cockatoos feeding on the (exceptionally large) prerelease seeds] were occasionally observed but removal of entire cones was not. The cones are more difficult to remove as they are held deep within the crown (cauliflory) and are not as rewarding (to birds), usually comprising only one or two follicles per cone. He et al. [15] also proposed that the prevailing winds might be sufficient to lift and sweep seeds of *B. attenuata* and *B. hookeriana* to adjacent dunes: in *B. candolleana* four immigrants travelled less than 500 m to the adjacent dune and could fit into this category (Fig. 1). However, the prominent wing of *B. candolleana* seeds is easily dislodged (unlike the other two species) when obstacles are encountered, making entrainment (the seeds lie flat on the soil) and carriage less likely. This leaves uplift by wind vortices and horizontal drift back to earth by the prevailing winds as the main transport mechanism. The strong NW-NE trend in dispersal direction evident for *B. attenuata* [14], consistent with the dominant direction of prevailing winds in autumn, was not clearly demonstrated for *B. candolleana*, suggesting that the pattern was essentially controlled by the random direction of wind vortices. For all species, however, our detection of population connectivity reveals the existence of vectors with capacity for secondary dispersal of seeds across a heterogeneous landscape matrix.

The much lower rate of LDD of *B. candolleana* seeds compared with the other two species may explain its lower dune occupancy rate and population density (Table 4). Both could be expressions of a delay in colonization (Fig. 2), though the latter might be more a function of longer time to maturation and/or lower seed production and recruitment efficiency. Time to maturation and seed production show only minor differences between these clonal and non-clonal resprouters but the 4× greater longevity and 5× lower annual mortality rate of the former more than counter the 9× reduction in its recruitment rate (Table 5). Metapopulation modelling (e.g. as done in [46]) could further determine the overall importance of migration in accounting for population viability of this species.

The dunes have been stable for ∼20,000 years [50] and so would have been subject to many bouts of colonization by, and extinction of, *B. candolleana* during that time. The three aged populations are 0.4−2.3 km apart (Fig. 1), similar to the distances we recorded as the range of long distance seed dispersal, and were apparently colonized by *B. candolleana* 500, 700 and 950 y ago based on extrapolation of the best fit curves to one (Fig. 2). Though the population with oldest plants (three at 840 y) is considerably younger than the estimated maximum longevity of clones (1200 y) there is some possibility that it may represent a much older population (>1200 years) because sampling may have missed the oldest plants or they may have died prematurely. This possibility is unlikely for the two youngest populations. Even if the oldest plants are missing, the right-hand slopes of the distribution curves of the extant clones are preserved and extrapolation to one (the colonizer) can still approximate the time of colonization. However, the oldest plants sampled differed in observed age from the extrapolated age by only 5−100 years indicating that little extrapolation was in fact required. A complete shift of the slope to the left, due to massive death of clones older than the median in that population giving a false age of the oldest clone by extrapolation, seems highly unlikely. First, this species has extremely stable population dynamics (Table 4) so that mortality of established plants is negligible, even when subject to frequent fire. Second, we show that the dunes are matched environmentally and biologically (see Methods) so it begs the question why this wholesale shift would occur in one population but not in the others? This means that differences in growth rates also would not apply as an alternative explanation for differences in apparent population age. There is also a possibility that the oldest population has actually persisted over two or more generations, making it impossible to identify the time of colonization. This would mean that the rate of colonization is even slower than our estimates, barring extinction of earlier populations on these dunes. If any well-established prior population had been eliminated, it is likely this would have required a landscape-scale event, such as the most extreme of droughts, that would have exterminated all three populations.

A mean migration rate of <800 m in 200 years is a sobering statistic when contemplating the potential role of plant migration as a response to climate change [51]. Witkowski and Lamont [52] calculated that another clonal banksia, *B. goodii*, in the absence of LDD mechanisms (considered unlikely because of the forest-environment of this species), would traverse ∼200 m in 200 y and dismissed such a rate as able to contribute to the conservation of this species under projected climate change rates. Interestingly, He et al [15] estimated that a combination of exceptionally wet years (50% more than the mean) and a mean 13-year fire interval would enable *B. hookeriana* to recruit and ‘creep’ across the occasional bridges (elevated swales) between adjacent dunes every 200 y without needing to invoke LDD. The intermediary plants would die out once drier conditions returned. It is unclear if such a mechanism would be effective for *B. candolleana,* as it takes 10× as long to mature, though this species appears more tolerant of shallow soils than *B. hookeriana* [B. Lamont, pers. observ.].

How can the relatively low effective LDD rate of *B. candolleana* be explained? It cannot be due to its extremely low recruitment rate as dispersal rate is a set fraction of recruitment no matter what level of recruitment occurs. In the absence of any reason to believe that immigrants are less competitive (but see below), whenever recruitment occurs, immigrants among *B. candolleana* juveniles will make up only a third as many individuals as among *B. attenuata* and *B. hookeriana* juveniles. It also cannot be due to the extreme longevity of *B. candolleana* adults, as a set fraction of adults comprises immigrants independent of their longevity. In the absence of any reason to believe that immigrants are shorter lived at any point in time or at any particular life history stage, immigrants among *B. candolleana* adults will make up a third as many individuals as among *B. attenuata* and *B. hookeriana* adults.

*Banksia candolleana* has exceptionally large seeds – the largest palatable seeds in the study area apart from the extremely bitter and cryptic seeds of *Xylomelum angustifolium* (Proteaceae). Large seeds and seedlings are often more visible and attractive to granivores and herbivores [53], [54]. As such, seeds of *B. candolleana* may be favoured by granivores. In support, many husks of *B. candolleana* seeds, with their embryos removed by granivorous birds or rodents, were noted after fire in the study area [B. Lamont, pers. observ.]. This might explain why recruitment efficiency is so low in this species (only 1 in 5000 seeds) despite its exceptionally large seeds. It might also affect their relative contribution to the population once established, if seeds are more apparent having alighted anywhere on the soil surface rather than being swept into and buried in the (highly competitive) litter microsites soon after release [55] as proposed by He *et al*. [14] for *B. attenuata* to explain the reverse (i.e. why immigrants are successful).

Relative recruitment success is a function of seed size in this environment, as seeds provide an essential source of mineral nutrients for root growth [56], [57], [58]. It is possible that smaller seeds of *B. candolleana* are more buoyant than larger ones (and therefore more likely to become immigrants but less fit overall), but terminal velocity is not a simple function of seed size (Table 4, [55]), and even the smallest seeds of *B. candolleana* are much larger than those of *B. attenuata* (Table 4). While relative buoyancy has little explanatory value in *B. candolleana* LDD, relative entrainment might. The follicles are the largest in the genus, so that the seed must be lifted 5−6 cm to escape, and they are often pushed up against other stems, so that seed release is not efficient. The average location of follicles is a mere 20 cm above the ground, one-fifth the height of the other two banksias (Table 5). In addition, the winged plate that separates the two seeds and carries them with it as it dislodges from the follicle usually remains attached to the seeds and they fall as a unit to the ground with one side trapping one of the seeds beneath it (this does not occur with the other two species, B. Lamont, pers. observ.). Further, the cones are overtopped by a dense cover of twigs even postfire, 2−4× that of the other two species. In addition, the large wing is soon broken off once reaching the ground, giving less surface area relative to mass for uplift and entrainment. Thus, not only are there considerable obstacles to exposing the seeds to free air, but the critical wind speed for entraining the seeds must be achieved at or near ground level. All these aspects imply that seeds of *B*. *candolleana* are more likely than seeds of *B. attenuata* or *B. hookeriana* to collect within and around the maternal plant and are less likely to become airborne.

We have shown that a species with typical reproductive attributes of the clonal life-form (albeit unusually large seeds) has poor LDD seed properties compared with two congeneric co-occurring non-clonal species, despite essentially the same mode of dispersal and physical landscape context, and that these appear to account for its relatively low rates of LDD. Whether these dispersal attributes are idiosyncrasies of this species or to be expected of long-lived clonal species generally (where the imperative for extensive dispersal via seeds is relaxed) is worthy of further study. For example, in another co-occurring clonal banksia, *B. elegans*, despite prolific flowering, seed set is usually negligible so that LDD is also negligible [59], [60]. In this regard, there is no substitute for a thorough knowledge of the reproductive biology of the species under study so that taxon-specific attributes can assist interpretation of estimates of population connectivity from genetic data [16]. In addition, the findings suggest that LDD rates of resprouting species might vary as much within the group, depending on whether they are clonal or not, as between resprouters and nonsprouters. As our ability to genotype individuals for population genetic studies becomes increasingly feasible [61], so will the accuracy and power to better reveal the tail of the dispersal curve. When combined with detailed demographic studies on co-occurring species within model metapopulation systems such as ours, landscape genetic approaches will increasingly reveal the effects of life-history variation and landscape patterns on LDD and landscape connectivity.
